# The loss of NKX3.1 expression in testicular – and prostate – cancers is not caused by promoter hypermethylation

**DOI:** 10.1186/1476-4598-4-8

**Published:** 2005-02-03

**Authors:** Guro E Lind, Rolf I Skotheim, Mario F Fraga, Vera M Abeler, Rui Henrique, Fahri Saatcioglu, Manel Esteller, Manuel R Teixeira, Ragnhild A Lothe

**Affiliations:** 1 Department of Genetics, Institute for Cancer Research, The Norwegian Radium Hospital, 0310 Oslo, Norway; 2 Cancer Epigenetics Laboratory, the Spanish National Cancer Centre (CniO), Madrid, Spain; 3 Deparment of Pathology, Institute for Cancer Research, The Norwegian Radium Hospital, 0310 Oslo, Norway; 4 Department of Pathology, Portuguese Oncology Institute, Porto, Portugal; 5 Department of Molecular Biosciences, Institute of Biology, University of Oslo, Oslo, Norway; 6 Department of Genetics, Portuguese Oncology Institute, Porto, Portugal

## Abstract

**Background:**

Recent studies have demonstrated that the NKX3.1 protein is commonly down-regulated in testicular germ cell tumors (TGCTs) and prostate carcinomas. The homeobox gene *NKX3.1 *maps to chromosome band 8p21, which is a region frequently lost in prostate cancer, but not in TGCT. Mutations have not been reported in the *NKX3.1 *sequence, and the gene is hypothesized to be epigenetically inactivated. In the present study we examined the methylation status of the *NKX3.1 *promoter in relevant primary tumors and cell lines: primary TGCTs (n = 55), intratubular germ cell neoplasias (n = 7), germ cell tumor cell lines (n = 3), primary prostate adenocarcinomas (n = 20), and prostate cancer cell lines (n = 3) by methylation-specific PCR and bisulphite sequencing.

**Results and Conclusions:**

Down-regulation of NKX3.1 expression was generally not caused by promoter hypermethylation, which was only found in one TGCT. However, other epigenetic mechanisms, such as modulation of chromatin structure or modifications of histones, may explain the lack of NKX3.1 expression, which is seen in most TGCTs and prostate cancer specimens.

## Background

The protein expression of the homeobox gene *NK3 transcription factor related locus 1 (NKX3.1) *is highly specific for the prostate and the testis [1-3], and is frequently lost in cancers of these two tissue types [1,4,5]. *NKX3.1 *is located in chromosome band 8p21 [2,6,7], a region that undergoes frequent allelic imbalance in prostatic intraepithelial neoplasia (PIN) and prostate carcinomas [8,9]. In mice, targeted disruption of *Nkx3.1 *leads to prostatic epithelial hyperplasia and dysplasia [10,11], and over-expression of exogenous *NKX3.1 *suppresses growth and tumorigenicity in human prostate carcinoma cell lines [12]. However, the expression levels and possible role for NKX3.1 during prostate cancer progression in humans is still being debated [13-15]. No gene mutations of *NKX3.1 *have been found [6], and *NXK3.1 *is therefore believed to be epigenetically inactivated in the cases with loss of protein expression [1,5,16]. Only one study has reported NKX3.1 protein expression in testicular germ cell tumors (TGCTs), however the series analyzed was large, including a total of more than 500 samples, and NKX3.1 was found absent in all embryonal carcinomas and present in only 15–20% of the seminomas as well as among the differentiated histological subtypes of germ cell tumors [5].

During the last decade, epigenetic changes in cancer have been frequently reported and are now recognized to be at least as common as genetic changes [17]. The best characterized epigenetic mechanism is DNA hypermethylation, in which cytosines located within selected CpG sites in the gene promoters become methylated, thereby inactivating gene expression. Several tumor suppressor genes are inactivated by such promoter hypermethylation in various cancer types [18,19]. In the present study we have performed methylation-specific PCR (MSP) and bisulphite sequencing of the *NKX3.1 *promoter in TGCTs and prostate adenocarcinomas to examine whether this mechanism may explain the commonly observed loss of NKX3.1 protein.

## Results

**Figure 1:**
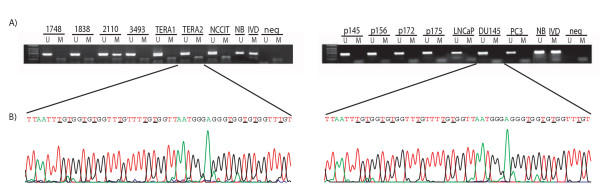
**Representative results of the methylation analyses of the *NKX3.1 *promoter. **(A) Methylation-specific PCR. A visible PCR product in *Lanes U *indicates the presence of unmethylated alleles whereas a PCR product in *Lanes M *indicates the presence of methylated alleles. The left panel illustrates the methylation status of selected TGCTs and the testicular cancer cell lines. Note the methylation of sample # 2110. The right panel shows the unmethylated status of primary prostate cancers and prostate cancer cell lines. Abbreviations: *NB*, normal blood (positive control for unmethylated samples); *IVD*, *in vitro *methylated DNA (positive control for methylated samples); *neg*, negative control (containing water as template); *U*, lane for unmethylated MSP product; *M*, lane for methylated MSP product. (B) Bisulphite sequencing. The bisulphite sequence allows a positive display of 5-methyl cytosines in the gene promoter as unmethylated cytosines appear as thymines, while 5-methylcytosines appear as cytosines in the final sequence. The left chromatogram represents a part of the unmethylated *NKX3.1 *promoter in the germ cell tumor cell line TERA2, including 11 CpG sites marked by underlined letters. The right chromatogram represents the unmethylated prostate cancer cell line DU145. Both sequences have been generated by reversing the respective anti-sense sequences by use of the software "Chromas".

Only one out of 54 TGCTs and none of the prostate adenocarcinomas (n = 20), intratubular germ cell neoplasias (n = 7), normal testis tissues (n = 4), or the cell lines (n = 6) displayed methylation when analyzed with MSP (Figure 1a). Bisulphite genomic sequencing of the tumors and cell lines showed that all cytosines at non-CpG sites were converted to thymine (Figure 1b). Only one sample demonstrated overall methylation in the *NKX3.1 *sequence, and this was the same sample that was positive for methylation from the MSP analysis. Interestingly, all the samples that were sequenced, including the normal blood, unmethylated cell lines, and primary tumors, displayed some extent of methylation (the majority below 25%) at the cytosine in CpG number 21 (base 1914762, +1 bp from transcription start). We detected a possible polymorphism in base 1914730 (+33 bp from transcription start and 15 bp upstream of the coding sequence). In previous sequences this site has been described as a guanine (Gene bank accession number NT_023666, and AF24770). In the cell lines, 5/6 contained adenosine in this position, but all except the germ cell tumor cell lines NCCIT and TERA2 were heterozygotes. In contrast, all 5 primary tumors sequenced were homozygous for the adenosine allele.

## Discussion

We have previously reported that the protein expression of NKX3.1 is virtually ubiquitously lost in TGCTs [5]. This was done using a tissue microarray containing 510 testicular tissue samples. NKX3.1 expression is known for 25 of the TGCTs now analyzed for promoter hypermethylation and 22 showed complete absence of protein. The down regulation of NKX3.1 in TGCT has also been detected at the mRNA level, both by quantitative RT-PCR [5] and from an oligonucleotide microarray study including 20 of the present TGCTs (Skotheim *et al*., submitted). This simultaneous down regulation of both protein and mRNA levels of *NKX3.1 *is consistent with epigenetic regulation, which is further supported by the fact that mutations have not been detected in the *NKX3.1 *gene [6]. DNA promoter hypermethylation is the best-characterized epigenetic change in cancer, and can be associated with gene silencing. It was therefore of interest to analyze the methylation status of the *NKX3.1 *promoter in TGCT and prostate cancer samples. However, with the exception of a single TGCT, the *NKX3.1 *promoter was unmethylated in the samples analyzed. The methylated TGCT was classified as a yolk sac tumor, and has also been demonstrated to have promoter hypermethylation of several other genes that are generally unmethylated in TGCTs (Lind *et al*., unpublished). We therefore consider this sample not to be representative for the general TGCT epigenotype, nor for the general epigenetic profile of yolk sac tumors. Thus, we do not regard promoter hypermethylation as the general mechanism of NKX3.1 down-regulation neither in TGCT nor in prostate carcinomas.

We also studied cell lines since it can be argued that presence of normal cells as well as tumor heterogeneity may mask cancer specific methylation in primary tumors. LNCaP cells have previously been demonstrated to express *NKX3.1*, in contrast to PC-3 and DU-145, which do not express NKX3.1 since they lack a functional androgen receptor [2]. The lack of *NKX3.1 *expression in PC-3 and DU-145 cells is not due to methylation. This was also the case with the germ cell tumor cell lines. From Western analysis, the cell line NCCIT had strong expression of NKX3.1 whereas both TERA1 and TERA2 had no expression (data not shown).

The polymorphism in *NKX3.1 *that we detected 15 bases upstream of the coding sequence has to our knowledge not been described previously. It was identified by bisulphite sequencing of the cell lines and a subgroup of primary tumors, thus caution should be taken when concluding from these results, since regular sequencing analysis is the recommended approach for describing sequence changes. As the polymorphism is located in the promoter region of *NKX3.1*, it has no influence on the protein structure. However, it can still have a potential role in the transcriptional regulation of *NKX3.1*. A polymorphism in the coding sequence is also reported for *NKX3.1 *[6].

All samples analyzed with bisulphite sequencing, including cell lines expressing NKX3.1, as well as non-expressing cell lines, demonstrated some degree of methylation in the cytosine in CpG number 21. As this site-specific methylation included only one CpG site, it is unlikely that it will have any regulating effect on gene expression. However, considering its intriguing location immediate upstream of the transcription start point, this possibility should not be excluded. There is also the possibility that the apparent methylation could be due to a less efficient bisulphite conversion for this site. In general, the bisulphite sequencing results showed that all cytosines at non-CpG sites were converted to thymine (Figure 1b), but sequence-specific partial resistance to this conversion may lead to methylation artifacts, but only in rare cases, as has been reported previously [20].

## Conclusions

In summary, these data show that the previously reported down-regulation of NKX3.1 in TGCTs and prostate carcinomas is not caused by promoter hypermethylation. Even though the *NKX3.1 *promoter is unmethylated, the simultaneous down-regulation of mRNA and protein levels in TGCTs and the absence of mutations still make other epigenetic mechanisms, such as modulation of chromatin structure or modifications of histones, possible explanations for loss of NKX3.1 expression in testicular- and prostate cancers.

## Materials and Methods

### Primary tumors and cell lines

Included in the present study are primary TGCTs (n = 55), intratubular germ cell neoplasias (also called carcinoma *in situ*; n = 7), normal testis tissue (n = 4), germ cell tumor cell lines (TERA1, TERA2, and NCCIT), prostate adenocarcinomas (n = 20), and prostate cancer cell lines (LNCaP, PC-3, and DU-145). The primary TGCTs include all histological subtypes: seminomas, embryonal carcinomas, teratomas, yolk sac tumors, and one choriocarcinoma, classified according to the WHO's recommendations [21] by a germ cell tumor reference pathologist using light microscopic examination of hematoxylin and eosin stained tissue sections. From our previous comparative genome hybridization analysis, about half of the TGCTs had a low-level copy number gain at chromosome 8, but only rarely 8p deletions [22]. Primary prostate adenocarcinomas obtained from radical prostatectomy specimens were graded according to the Gleason grading system [23] using routinely stained tissue sections. The median Gleason score of prostate adenocarcinomas was 7 (range: 4 – 8). The prostate carcinomas were all of pTNM stage 2 and 3, and included 10 samples with 8p deletions (among other cytogenetic aberrations), 3 samples with copy number changes not involving the 8p region, and 7 samples with no copy number changes (Ribeiro *et al*., submitted).

### Methylation-specific PCR

The DNA samples were initially bisulphite modified [24,25], which converts unmethylated but not methylated cytosines to uracil. All samples were subsequently submitted to MSP analysis [26] using PCR primers specific to methylated and unmethylated sequences: *NKX3.1 *unmethylated sequence, sense: 5'GGAAAGTGAAAGTGGTGTGGGTT3', antisense: 5'CTACACACCATCCCACAAAATATC3', methylated sequence, sense: 5'AAAGTGAAAGCGGTGCGGGTC3', antisense: 5'ACGCGCCGTCCCGCAAAATAT3' (MedProbe AS, Oslo, Norway). The two fragments were amplified by the Fast Star DNA polymerase (Roche Ltd, Basel, Switzerland) in a reaction containing 1.5 mM Mg^2+^. We used a 58°C annealing temperature for both primer sets. Bisulphite treated DNA from normal blood (NB) and *Sss1 *methyltransferase (New England Biolabs Inc., Beverly, MA, USA) *in vitro *treated placenta DNA (IVD; Sigma Chemical Co., St. Louis, MO, USA) represented the unmethylated positive control and the methylated positive control, respectively. Water, replacing bisulphite treated template, was the negative control in both reactions.

### Bisulphite sequencing

Bisulphite sequencing allows a positive display of 5-methyl cytosines in the gene promoter after bisulphite modification as unmethylated cytosines appear as thymines, while 5-methylcytosines appear as cytosines in the final sequence [27]. A subset of the samples (n = 11) were bisulphite sequenced, including all 6 cell lines, 3 TGCTs, and 2 prostate adenocarcinomas. Additionally, NB and IVD were bisulphite sequenced as positive controls for unmethylated and methylated sequence, respectively. The *NKX3.1 *bisulphite sequence fragment (Gene bank accession number NT_023666 (minus strand), bases 1914526 to 1914961) was 436 bp long and covered 52 CpG sites in the promoter and first exon of the gene. We designed bisulphite sequencing primers (MedProbe) with the following sequences; sense: 5'ATTGGGGAAGGAGAGGGAATTG3', antisense: 5'CCTCTAACTCTAACTCTAACTCC3'. The Mg^2+ ^content of the reaction was 1.3 mM, the enzyme used was HotStarTaq™ DNA polymerase (QIAGEN Inc., Valencia, CA, USA), and the annealing temperature 52°C. The PCR fragments were eluted from a 2% agarose gel (BioRad Laboratories Inc, CA, USA) containing ethidium bromide, by the MinElute™Gel Extraction kit (QIAGEN), and sequenced with the dGTP BigDye Terminator Cycle Sequencing Ready Reaction kit (Applied Biosystems, Foster City, CA) in an ABI Prism 377 Sequencer (Applied Biosystems). The bisulphite sequencing results were scored according to Melki *et al*. where the amount of methylcytosine of each CpG dinucleotide is quantified by comparing the peak height of the cytosine signal with the sum of the cytosine and thymine peak height signals [28].

## Authors' contributions

GEL performed the experimental analyses and statistics, interpreted the results, and drafted the manuscript. RIS did an independent scoring of the results, and contributed to manuscript preparation. MFF designed the primers used for the MSP and bisulphite treatment and contributed to manuscript preparation. VMA and RH were reference pathologists for the testicular cancer tissues and prostate tissues, respectively. FS was responsible for the Western Blot studies of the cell lines and participated in the writing of the manuscript. Parts of this work were done in the lab of ME who also contributed with scientific discussions. MRT provided the relevant selected series of primary prostate carcinomas with known genetic profiles, and contributed to manuscript preparation. RAL conceived the study, was responsible for its design and coordination, and contributed in the evaluation of the results and in preparation of the manuscript.
